# Omega, Sadhana, and PI Polynomials of Quasi-Hexagonal Benzenoid Chain

**DOI:** 10.1155/2020/9057815

**Published:** 2020-03-26

**Authors:** Nazeran Idrees, Muhammad Jawwad Saif, Sumiya Nasir, Fozia Bashir Farooq, Asia Rauf, Fareeha Ashfaq

**Affiliations:** ^1^Department of Mathematics, Government College University Faisalabad, 38000 Faisalabad, Pakistan; ^2^Department of Applied Chemistry, Government College University Faisalabad, 38000 Faisalabad, Pakistan; ^3^Core Curriculum, College of Science and Human Studies, Prince Mohammad Bin Fahd University, 34754 Khobar Dhahran, Saudi Arabia; ^4^Department of Mathematics, Al Imam Mohammad Ibn Saud University, 11432 Riyadh, Saudi Arabia; ^5^Department of Mathematics, Government College Women University Faisalabad, 38000 Faisalabad, Pakistan

## Abstract

Counting polynomials are important graph invariants whose coefficients and exponents are related to different properties of chemical graphs. Three closely related polynomials, i.e., Omega, Sadhana, and PI polynomials, dependent upon the equidistant edges and nonequidistant edges of graphs, are studied for quasi-hexagonal benzenoid chains. Analytical closed expressions for these polynomials are derived. Moreover, relation between Padmakar–Ivan (PI) index of quasi-hexagonal chain and that of corresponding linear chain is also established.

## 1. Introduction

Counting polynomials are a well-known way of expressing molecular invariants of a chemical graph in polynomial form. These polynomials depend on chemical graph properties such as matching sets, independent sets, chromatic numbers, and equidistant edges. Some well-known polynomials are Hosoya polynomial, Wiener polynomial, sextet polynomial, matching polynomial, and chromatic polynomials. Many important topological indices can be derived from polynomials by directly taking their value at some point or after taking derivatives or integrals. Topological index is a numeric quantity related to a graph which predicts the chemical properties, physical properties, and biological activity. These invariants are used in chemical modeling, drug designing, and structural activity relations. Counting polynomial is generally expressed as

(1) $$\begin{array}{c} P\left(G,x\right)=\underset{k}{\sum }m\left(G,k\right)\times x^{k}, \end{array}$$

where *k* is the extent of a property partition and coefficients *m*(*G*, *k*) denote the multiplicity of the partition. The corresponding topological index *P*(*G*) is derived as follows:

(2) $$\begin{array}{c} P\left(G\right)=P^{\prime }{\left.\left(G,x\right)\right|}_{x=1}. \end{array}$$

These polynomials count equidistant and nonequidistant edges in a graph and are very important in prediction of physiochemical properties of a molecule.

Let *G*=(*V*, *E*) be a connected graph with the vertex set *V*=*V*(*G*) and the edge set *E*=*E*(*G*), without loops and multiple edges. A molecular/chemical graph is a simple finite graph in which vertices denote the atoms and edges denote the chemical bonds in the underlying chemical structure. The hydrogen atoms are often omitted in any molecular graph. A chemical graph can be represented by a matrix, a sequence, a polynomial, and a number (often called a topological index) which represents the whole graph, and these representations are aimed to be uniquely defined for that graph. Two edges *e*=*ab* and *f*=*cd* of a graph *G* are said to be codistant, usually denoted by *eCof*, if

(3) $$\begin{array}{c} d\left(a,c\right)=d\left(b,d\right)\text{ and }d\left(a,d\right)=d\left(b,c\right)=d\left(a,c\right)+1=\;d\left(b,d\right)+1,\\ eCoe,\\ eCof⟺fCoe. \end{array}$$

“*Co*” is reflexive and symmetric but may not be transitive. Consider *C*(*e*)={*f* ∈ *E*(*G*) : *fCoe*}.

If the relation is transitive on *C*(*e*) also, then *C*(*e*) is called an orthogonal cut (OC) of the graph *G*, and if the relation is not transitive then it is termed as quasi-orthogonal cut (QOC). Let *e* = *ab* and *f* = *cd* be two edges of a graph *G*, which are opposite or topologically parallel, and this relation is denoted by *eopf*. A set of opposite edges, within the same face or ring, eventually forming a strip of adjacent faces/rings, is called an opposite edge strip (OPS), which is a quasi-orthogonal cut (QOC).

Omega, Sadhana, and PI polynomials are defined on the basis of quasi-orthogonal cuts. Let  *m*(*G*,  *k*) denote the number of QOCs of length *k* and *e*=|*E*(*G*)| is number of edges of *G*.

The Omega polynomial was introduced by Diudea [1] denoted by Ω(*G*, *x*) and is defined as

(4) $$\begin{array}{c} Ω\left(G,\;x\right)=\underset{k}{\sum }m\left(G,x\right)\times x^{k}. \end{array}$$

The Sadhana polynomial was proposed by Ashrafi et al. [2] and is defined as

(5) $$\begin{array}{c} Sd\left(G,x\right)=\underset{k}{\sum }m\left(G,k\right)\times x^{e-k}. \end{array}$$

Khadikar et al. introduced a remarkable topological index called Padmakar–Ivan (PI) index [3]. Ashrafi et al. [4] introduced the PI polynomial based on counting opposite edge strips in any graph. This polynomial counts nonequidistant edges in *G*, denoted by PI(*G*, *x*), and is defined as

(6) $$\begin{array}{c} \text{PI}\left(G,x\right)=\underset{k}{\sum }m\left(G,k\right)\times k\times x^{e-k}. \end{array}$$

Counting polynomials have been a subject of interest for researchers working in chemical graph theory, some recent work included in [2–8]. Moreover, different topological aspects of hexagonal chains have been studied in [9–12].

In this paper, we aim to find counting polynomials, i.e., Omega polynomial, Sadhana polynomial, and PI polynomial of quasi-hexagonal chain. These three polynomials depend upon the distance between the edges of chemical graphs. Moreover, topological invariants of the quasi-hexagonal benzenoid chain related to these counting polynomials such as Sadhana index and Padmakar–Ivan (PI) index are also analysed. Relation between the PI index of the quasi-hexagonal chain and linear hexagonal chain is also established.

## 2. Results and Discussions

### 2.1. Quasi-Hexagonal Chain and Counting Polynomials

**Figure 1:**
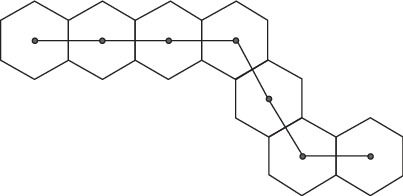
A quasi-hexagonal chain *H*_7_ and its inner duals.

A chain is said to be quasi-hexagonal chain if it can be embedded into the normal hexagonal cross section/lattice in the plane without overlapping of its vertices. A quasi-hexagonal chain, symbolized as *H*_*n* _, with *n* hexagons can be defined inductively as follows: a quasi-hexagonal chain *H*_1_ is a hexagon. For *n* > 1, a quasi-hexagonal chain *H*_*n* _ is acquired from a quasi-hexagonal chain *H*_*n*−1 _ by appending another hexagon to an end hexagon at one of its sides on the limit of *H*_*n*−1_.  Figure 1 shows an example of a quasi-hexagonal chain. These chains correspond to various benzenoid systems and provide a general way to express these systems in the form of a chemical graph. Some aromatic hydrocarbons, for example, dibenz[a,h] anthracene, with five fused benzene rings, provide an example of quasi-hexagonal chains. Moreover, naphthalene, anthracene, chrysene, and phenanthrene ring systems are some other instances of quasi-hexagonal chains.

A quasi-hexagonal chain has segments consisting of maximal linear chains. The number of vertices in the inner dual of each segment is called its length. It can be denoted by *H*(*l*_1_, *l*_2_,…, *l*_*s*_), where *s* denotes the number of segments in the chain and *l*_*i*_ denote the length of each segment. (*l*_1_, *l*_2_,…, *l*_*s*_) is termed as the length vector of the quasi-hexagonal chain. Moreover, it can be noted that ∑_1_^*s*^*l*_*i*_=*n*+*s* − 1, where *n* denotes the total number of hexagons in the chain and *s* denotes the number of segments of the chain.

The total number of edges in quasi-hexagonal is |*E*|=5∑_1_^*s*^*l*_*i*_ − 5*s*+6. If the inner dual of a quasi-hexagonal chain is linear, then the chain is called a linear chain.

**Figure 2:**
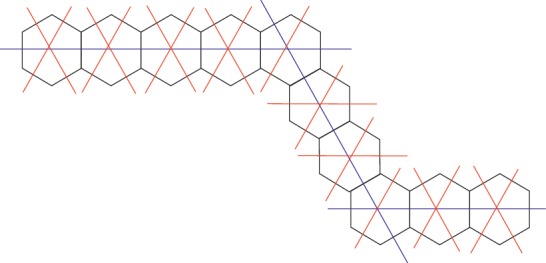
The elementary cuts of the quasi-hexagonal chain *H*_10_(5,4,3).

We will find distance-based polynomials by using the edge-cut method introduced by Klavzar [13]. The cut lines of *H*_*n* _ are divided into two classes: (i) they intersect only one hexagonal, and they are orthogonal to the same edge direction; (ii) they intersect at least one hexagonal, and they are orthogonal to the remaining two edge directions. The former are denoted by red cuts and the later are denoted by blue cuts (see Figure 2).

The number of quasi-orthogonal cuts which intersect only one hexagon (having only two codistant edges) is equal to 2∑_1_^*s*^*l*_*i*_ − 3(*s* − 1). The second type of QOCs cut each segment once and has a length equal to *l*_*i*_+1, for each *i*=1,2,…, *s*. These are summarized in Table 1.

**Theorem 1 .**

The Omega polynomial of the quasi-hexagonal chain *H*_*n* _ with length vector (*l*_1_, *l*_2_,…, *l*_*s*_) is as follows:

(7) $$\begin{array}{c} Ω\left(H_{n\;},x\right)=x\left(x^{l_{1}}+x^{l_{2}}+\ldots +x^{l_{s}}\right)+x^{2}\left(2n-s+1\right). \end{array}$$

**Proof**

Let *G* be a graph of the quasi-hexagonal chain *H*_*n* _. The Omega polynomial of graph *G* is given by equation (4) as Ω(*G*, *x*)=∑_*k*_*m*(*G*, *k*) × *x*^*k*^.

Now, using Table 1 for number of codistant edges and substituting the values of quasi-orthogonal cuts and number of cuts, we get

(8) $$\begin{array}{c} Ω\left(H_{n\;},x\right)=1.x^{l_{1}+1}+1.x^{l_{2}+1}+\cdots +1.x^{l_{s}+1}+\left(2\overset{s}{\underset{1}{\sum }}l_{i}-3\left(s-1\right)\right)x^{2}=x^{l_{1}+1}+x^{l_{2}+1}+\cdots +x^{l_{s}+1}+2x^{2}\overset{s}{\underset{1}{\sum }}l_{i}-3\left(s-1\right)x^{2}\\ =x^{l_{1}}.x+x^{l_{2}}.x+\cdots +x^{l_{s}}.x+2x^{2}\left(n+s-1\right)-3\left(s-1\right)x^{2}, \end{array}$$

where

(9) $$\begin{array}{c} \sum_{1}^{s}l_{i}=\left(n+s-1\right)\\ =x\left(x^{l_{1}}+x^{l_{2}}+\cdots +x^{l_{s}}\right)+x^{2}\left(2n-2+2s-3s+3\right). \end{array}$$

By simplifying, we get the Omega polynomial of *H*_*n* _ as follows:

(10) $$\begin{array}{c} Ω\left(H_{n\;},x\right)=x\left(x^{l_{1}}+x^{l_{2}}+\cdots +x^{l_{s}}\right)+x^{2}\left(2n-s+1\right). \end{array}$$

**Theorem 2 .**

The Sadhana polynomial of the quasi-hexagonal chain *H*_*n*_ is given by

(11) $$\begin{array}{c} Sd\left(H_{n},x\right)=x^{5n}\left(x^{-l_{1}}+x^{-l_{2}}+\cdots +x^{-l_{s}}\right)+x^{5n-1}\left(2n-2s+1\right). \end{array}$$

**Proof**

Consider graph *G* of the quasi-hexagonal chain *H*_*n* _. The Sadhana polynomial is defined as follows:

(12) $$\begin{array}{c} Sd\left(G,x\right)=\underset{k}{\sum }m\left(G,k\right)\times x^{e-k},\;e=\left|E\right|=5\overset{s}{\underset{1}{\sum }}l_{i}-5s+6. \end{array}$$

Substituting the values of codistant edges and number of quasi-orthogonal cuts from Table 1, we obtain

(13) $$\begin{array}{c} Sd\left(H_{n},x\right)=1.x^{5\sum_{1}^{s}l_{i}-5s+6-l_{1}-1}+1.x^{5\sum_{1}^{s}l_{i}-5s+6-l_{2}-1}+\cdots +1.x^{5\sum_{1}^{s}l_{i}-5s+6-l_{s}-1}+\left(2\overset{s}{\underset{1}{\sum }}l_{i}-3\left(s-1\right)\right)x^{5\sum_{1}^{s}l_{i}-5s+6-2}\\ =1.x^{5\sum_{1}^{s}l_{i}-5s+5-l_{1}}+1.x^{5\sum_{1}^{s}l_{i}-5s+5-l_{2}}+\cdots +1.x^{5\sum_{1}^{s}l_{i}-5s+5-l_{s}}+\left(2\overset{s}{\underset{1}{\sum }}l_{i}-3\left(s-1\right)\right)x^{5\sum_{1}^{s}l_{i}-5s+4}\\ =x^{5\sum_{1}^{s}l_{i}-5s+5}\left(x^{-l_{1}}+x^{-l_{2}}+\cdots +x^{-l_{s}}\right)+\left(2\overset{s}{\underset{1}{\sum }}l_{i}-3\left(s-1\right)\right)x^{5\sum_{1}^{s}l_{i}-5s+4}. \end{array}$$

Here, we substitute ∑_1_^*s*^*l*_*i*_=(*n*+*s* − 1) in the above expression and obtain

(14) $$\begin{array}{c} S\;d\left(H_{n},x\right)=x^{5\left(n+s-1\right)-5s+5}\left(x^{-l_{1}}+x^{-l_{2}}+\cdots +x^{-l_{s}}\right)+x^{5\left(n+s-1\right)-5s+4}\left(2\left(n+1-s\right)+3\left(s-1\right)\right). \end{array}$$

By simplifying, we get

(15) $$\begin{array}{c} S\;d\left(H_{n\;},x\right)=x^{5n}\left(x^{-l_{1}}+x^{-l_{2}}+\cdots +x^{-l_{s}}\right)+x^{5n-1}\left(2n-2s+1\right). \end{array}$$

**Remark 1 .**

Sadhana index, introduced by Khadikar et al. [14] while modeling aromatic stabilities of acenes and helicenes, is defined as the first derivative of the Sadhana polynomial at *x* = 1. The Sadhana index of the quasi-hexagonal chain *H*_*n* _ is given as

(16) $$\begin{array}{c} S\;d\left(H_{n\;}\right)=\frac{\text{d}}{\text{d}x}{\left(S\;d\left(H_{n\;},x\right)\right)}_{x=1}=10n^{2}-40sn+12n-24s-10. \end{array}$$

**Theorem 3 .**

Consider the quasi-hexagonal chain *H*_*n*_. Then, its PI index is given by

(17) $$\begin{array}{c} \text{PI}\left(H_{n\;},x\right)=\left(5n-1\right)\left(5n-s+1\right)-\overset{s}{\underset{1}{\sum }}l_{i}^{2}. \end{array}$$

**Proof**

Consider the graph *G* of the quasi-hexagonal chain *H*_*n* _:

(18) $$\begin{array}{c} \text{PI}\left(G,x\right)=\underset{k}{\sum }m\left(G,k\right)\times k\times x^{e-k}. \end{array}$$

Substituting the values in the above equation from Table 1, we obtain

(19) $$\begin{array}{c} \text{PI}\left(H_{n\;},x\right)=\left(l_{1}+1\right)x^{5\sum_{1}^{s}l_{i}-5s+6-l_{1}-1}+\left(l_{2}+1\right)x^{5\sum_{1}^{s}l_{i}-5s+6-l_{2}-1}+\cdots +\left(l_{s}+1\right)x^{5\sum_{1}^{s}l_{i}-5s+6-l_{s}-1}+\left(4\overset{s}{\underset{1}{\sum }}l_{i}-6\left(s-1\right)\right)x^{5\sum_{1}^{s}l_{i}-5s+6-2}\\ =\left(l_{1}+1\right)x^{5\sum_{1}^{s}l_{i}-5s+5-l_{1}}+\left(l_{2}+1\right)x^{5\sum_{1}^{s}l_{i}-5s+5-l_{2}}+\cdots +\left(l_{s}+1\right)x^{5\sum_{1}^{s}l_{i}-5s+5-l_{s}}+\left(4\overset{s}{\underset{1}{\sum }}l_{i}-6\left(s-1\right)\right)x^{5\sum_{1}^{s}l_{i}-5s+4}. \end{array}$$

Substituting ∑_1_^*s*^*l*_*i*_=*n*+*s* − 1, we obtain

(20) $$\begin{array}{c} \text{PI}\left(H_{n},x\right)=\left(l_{1}+1\right)x^{5n-l_{1}}+\left(l_{2}+1\right)x^{5n-l_{2}}+\cdots +\left(l_{s}+1\right)x^{5n-l_{s}}+\left(4n-2s+2\right)x^{5n-1}. \end{array}$$

By differentiating the above equation, we obtain

(21) PI'Hn,x=l1+15n−l1x5n−l1−1+l2+15n−l2x5n−l2−1+⋯+ls+15n−lsx5n−ls−1+4n−2s+25n−1x5n−2.

To compute the PI index of the quasi-hexagonal chain, we evaluate its value at *x*=1:

(22) PI'Hn,xx=1=l1+15n−l1+l2+15n−l2+⋯+ls+15n−ls+4n−2s+25n−1,PI'Hn,xx=1=5n−1∑i=1sli−∑1sli2+5n−14n−2s+2,PIHn=5n−1n+s−1−∑1sli2+5n−14n−2s+2,

where we have ∑_1_^*s*^*l*_*i*_=*n*+1 − *s*. Then,

(23) $$\begin{array}{c} \text{PI}\left(H_{n}\right)=\left(5n-1\right)\left(n+s-1+4n-2s+2\right)-\overset{s}{\underset{1}{\sum }}l_{i}^{2}. \end{array}$$

By simplifying, we get the PI index of *H*_*n* _ which is

(24) $$\begin{array}{c} \text{PI}\left(H_{n}\right)=\left(5n-1\right)\left(5n-s+1\right)-\overset{s}{\underset{1}{\sum }}l_{i}^{2}. \end{array}$$

### 2.2. Relation between the PI Index of the Linear Hexagonal Chain *L*_*n*_ and Quasi-Hexagonal Chain *H*_*n*_

**Figure 3:**
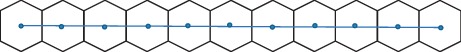
Linear hexagonal chain *L*_11_.

Let *L*_*n*_ denote the linear hexagonal chain as depicted in Figure 3. Quasi-orthogonal cuts and number of edges in these cuts are described in Table 2.

The following theorem gives the Padmakar–Ivan (PI) index of the linear hexagonal chain.

**Theorem 4 .**

Consider the graph of linear hexagonal chain *L*_*n* _. Then, its PI Index is given by

(25) $$\begin{array}{c} \text{PI}{\left.\left(L_{n}\right)\right|}_{x=1}=24n^{2}. \end{array}$$

**Proof**

Let *G* be a graph of the linear hexagonal chain *L*_*n*_. We employ Table 2 to prove the theorem. We apply formula and do some computations to gain the solution. As we know by definition, the PI Index is given by equation (6) as

(26) $$\begin{array}{c} \text{PI}{\left.\left(L_{n},x\right)\right|}_{x=1}=\underset{k}{\sum }m\left(L_{n},k\right)\times k\times x^{e-k}, \end{array}$$

Using Table 2,

(27) $$\begin{array}{c} \text{PI}\left(L_{n},x\right)=\left(n+1\right)x^{5n+1-\left(n+1\right)}+2n\left(2\right)x^{5n+1-2}\\ =\left(n+1\right)x^{4n}+4nx^{5n-1}. \end{array}$$

Differentiating with respect to *x*, we obtain

(28) PI'Ln,x=n+14nx4n−1+4n5n−1x5n−2.

Substituting *x*=1, we obtain the Padmakar–Ivan (PI) index of the linear hexagonal chain:

(29) $$\begin{array}{c} \text{PI}\left(L_{n}\right)=24n^{2}. \end{array}$$

**Theorem 5 .**

Consider a quasi-hexagonal chain (with *n* hexagons) *H*_*n*_, with *L*(*G*)=(*l*_1_, *l*_2_,…, *l*_*s*_), where *L*_*n*_  be the corresponding linear hexagonal chain. Then, PI(*H*_*n*_)=PI(*L*_*n*_)+*n*^2^ − ∑_*i*=1_^*s*^*l*_*i*_^2^+100*n* − 100*s*^2^ − 100*ns*+185*s*+89.

**Proof**

From Theorem 3, we can easily see that

(30) $$\begin{array}{c} \text{PI}\left(H_{n}\right)=\left(5n-1\right)\left(5n-s+1\right)-\overset{s}{\underset{1}{\sum }}l_{i}^{2},\\ \text{PI}\left(H_{n}\right)=25n^{2}-5n+s-1-\overset{s}{\underset{1}{\sum }}l_{i}^{2}. \end{array}$$

Moreover, from Theorem 4, we have

(31) $$\begin{array}{c} \text{PI}\left(L_{n}\right)=24n^{2}. \end{array}$$

Comparing (30) and (31), we get the desired relation between the PI index of the two chains:

(32) $$\begin{array}{c} \text{PI}\left(H_{n}\right)=\text{PI}\left(L_{n}\right)+24n^{2}-5n+s-1-\overset{s}{\underset{1}{\sum }}l_{i}^{2}. \end{array}$$

Counting polynomials of the quasi-hexagonal chain *H*_*n*_ are analysed for different values of *n*, and the results are summarized in Table 3.

## 3. Concluding Remarks

Counting polynomials provide an elegant way to encode the topological indices of chemical graphs, which are quantifiers of different physiochemical properties of the compounds and are widely used in structure activity relationships. Distance-based counting polynomials, namely, Omega, Sadhana, and PI polynomials of the quasi-hexagonal benzenoid chain are computed via the edge-cut method of Klavzar. Quasi-hexagonal benzenoid chains are an abstract idea to present a wide class of aromatic hydrocarbons. These polynomials are well-known tools for correlating the chemical graph with the physiochemical properties of different benzenoid chains. Moreover, the numerical invariant of the linear hexagonal chain (of benzenoid systems) is derived and its relation with that of the quasi-hexagonal chain is established.

## Figures and Tables

**Table 1:** Number of codistant edges of the quasi-hexagonal chain *H*_*n*_.

Types of edges	Types of QOCs	No. of QOCs	No. of codistant edges
*t*_1_	*M*	1	*l*_1_+1
		1	*l*_2_+1
		—	—
		—	—
		—	—
		1	*l*_*s*_+1
*t*_2_	*S*	2∑_1_^*s*^*l*_*i*_ − 3(*s* − 1)	2

**Table 2:** Number of codistant edges of the linear hexagonal chain *L*_*n*_.

Types of edges	Types of QOCs	No. of QOCs	No. of codistant edges
*t*_1_	*m*_1_	1	*n*+1
*t*_2_	*m*_2_	2*n*	2

**Table 3:** Omega, Sadhana, and PI polynomials of the quasi-hexagonal chain for different values of *n*.

*n*		(*l*_1_, *l*_2_,…, *l*_*s*_)	Ω(*H*_*n*_, *x*)	*S*(*H*_*n*_, *x*)	PI(*H*_*n*_, *x*)
1	1	(1)	3*x*^2^	2*x*^4^	6*x*^4^
2	1	(2)	*x* ^3^+3*x*^2^	3*x*^9^+*x*^8^	8*x*^9^+3*x*^8^
3	1	(3)	*x* ^4^+6*x*^2^	5*x*^14^+*x*^12^	12*x*^14^+4*x*^12^
	2	(2, 1)	2*x*^3^+5*x*^2^	3*x*^14^+2*x*^13^	10*x*^14^+6*x*^13^
4	1	(4)	*x* ^5^+8*x*^2^	7*x*^19^+*x*^16^	16*x*^19^+5*x*^16^
	2	(3, 2)	*x* ^4^+*x*^3^+7*x*^2^	3*x*^19^+*x*^18^+*x*^17^	14*x*^19^+3*x*^18^+4*x*^17^
	3	(2, 2, 2)	6*x*^3^+6*x*^2^	3*x*^19^+3*x*^18^	12*x*^19^+9*x*^18^
5	1	(5)	11*x*^2^	10*x*^24^+*x*^20^	20*x*^24^+6*x*^20^
	2	(4, 2)	*x* ^4^+*x*^3^+9*x*^2^	7*x*^24^+*x*^23^+*x*^21^	18*x*^24^+3*x*^23^+5*x*^21^
	3	(3, 2, 2)	*x* ^4^+2*x*^3^+8*x*^2^	5*x*^24^+2*x*^23^+*x*^22^	16*x*^24^+6*x*^23^+*x*^22^
	4	(2, 2, 2, 2)	4*x*^3^+7*x*^2^	3*x*^24^+4*x*^23^	14*x*^24^+12*x*^23^
6	1	(6)	*x* ^7^+12*x*^2^	11*x*^29^+*x*^24^	20*x*^29^+6*x*^20^
	2	(5, 2)	*x* ^6^+*x*^3^+11*x*^2^	9*x*^29^+*x*^28^+*x*^25^	22*x*^29^+3*x*^28^+6*x*^25^
	3	(4, 2, 2)	*x* ^5^+2*x*^3^+10*x*^2^	7*x*^29^+2*x*^28^+*x*^26^	20*x*^29^+6*x*^28^+*x*^26^
	4	(3, 2, 2, 2)	*x* ^4^+3*x*^3^+9*x*^2^	5*x*^29^+3*x*^28^+*x*^27^	18*x*^29^+9*x*^28^+4*x*^27^

## Data Availability

The data used to support the findings of this study are included within the article.
